# Efficacy of manual acupuncture, electro-acupuncture, and warm acupuncture for knee osteoarthritis: study protocol for a randomized controlled trial

**DOI:** 10.1186/s13063-022-06653-7

**Published:** 2022-08-20

**Authors:** Yiniu Chang, Nan Wu, Zhenhua Zhang, Zhaoyang Zhang, Binbin Ren, Feilai Liu, Xiaolei Song, Mingli Wu, Xiaodong Feng, Shuai Yin

**Affiliations:** 1 Henan University of Chinese Medicine, No. 156 East Jinshui Road, Zhengzhou, Henan China; 2 Department of Rehabilitation, The First Affiliated Hospital of Henan University of Chinese Medicine, No. 19 Renmin Road, Zhengzhou, Henan China

**Keywords:** Knee osteoarthritis, Manual acupuncture, Electro-acupuncture, Warm acupuncture, Randomized controlled trial

## Abstract

**Background:**

Acupuncture is one of the most popular complementary and alternative treatments for knee osteoarthritis (KOA). There are many methods of acupuncture in the treatment of KOA, and the effects are different. According to our clinical observations and researches, it is found that manual acupuncture (MA), electro-acupuncture (EA), and warm acupuncture (WA) are used more frequently in the treatment of KOA, and the curative effects are satisfactory. However, there is currently a lack of efficacy comparison of efficacy between different acupuncture treatments, as well as a lack of standardized clinical research on the acupuncture treatment of KOA. Therefore, we will carry out a high-quality clinical randomized controlled trial to research the effect laws of MA, EA, and WA on KOA.

**Methods/design:**

A total of 200 eligible participants with KOA will be randomly assigned to group A, B, C, or D in a ratio of 1:1:1:1. Patients in group A will receive MA, while those in group B, group C, and group D will be treated with EA, WA, and sham acupuncture (SA), respectively. Patients will be treated with acupuncture once a day, 30 min per session, 5 sessions per week for 4 weeks. The primary outcome is the change of Western Ontario and McMaster Universities Osteoarthritis Index (WOMAC) at week 4. The secondary outcomes include WOMAC, visual analog scale (VAS), Arthritis Quality of Life Measurement Scale Simplified Scale (AIMS2-SF), Beck Anxiety Inventory (BAI), Beck Depression Inventory (BDI), and Credibility/Expectancy Questionnaire. The evaluation will be performed at baseline and weeks 4, 8, and 12 respectively after randomization.

**Discussion:**

This is a randomized controlled trial. We will observe the clinical effect of MA, EA, and WA on KOA to research the effect laws of these three acupuncture treatments on KOA and set up standardized treatment programs for acupuncture for KOA.

**Trial registration:**

China Clinical Trials Registry ChiCTR2100049526. Registered on August 2, 2021

**Supplementary Information:**

The online version contains supplementary material available at 10.1186/s13063-022-06653-7.

## Introduction

Knee osteoarthritis (KOA) is a common chronic progressive disease characterized by the degeneration and secondary bone hyperplasia of the knee articular cartilage. Pain and functional limitation are the primary clinical symptoms of KOA that prevent patients from engaging in their usual activities [1]. According to an epidemiological survey in the USA, about 240 people are diagnosed with KOA every year in every 100,000 people. Among people over 45 years old, the prevalence of KOA in males is about 6–13% and in females is about 7–19%, and females have a 45% higher risk than males [2]. The overall KOA prevalence in Spain, Italy, Greece, and Norway is 12.2%, 5.4%, 6.0%, and 7.1%, respectively [3]. In China, the incidence of KOA is 8.1%, of which 5.7% in men and 10.3% in women [4]. As a high-prevalence chronic joint disease [5], KOA has become one of the leading causes of disability in the world and brings an increasing social and economic burden [6]. With the aging population, KOA is now a major health risk for middle-aged and elderly people [7].

Patients with KOA often present joint pain, stiffness, and limited mobility. However, knee pain largely affects KOA patients’ quality of life and is the major reason patients seek medical help and advice [5]. The aim of KOA treatment can provide symptomatic pain relief and improve knee function and the quality of life [8]. Currently, drug therapy and surgical treatment are the two major interventions for KOA. Drug therapy takes effect very soon, but it always comes with certain side effects and gastrointestinal adverse reactions. Surgical treatment tends to be expensive and is often accompanied by contraindications and complications, limiting its wide application.

Considering the increasing burden of knee surgery and the side effects of conventional medications, an increasing number of patients with KOA are likely to choose complementary and alternative treatments [9]. As one of the most popular complementary and alternative treatments [10], acupuncture has been used to treat osteoarthritis disorders in China since ancient times [11], and systematic reviews of randomized controlled trials (RCTs) suggested the benefits of acupuncture for KOA [12, 13]. Moreover, many guidelines for the diagnosis and treatment of KOA also recommend using acupuncture in the treatment of KOA [14, 15]. However, there are many methods to treat KOA with acupuncture, and the curative effects are also different. Based on our clinical observations and researches, it is found that manual acupuncture (MA), electro-acupuncture (EA), and warm acupuncture (WA) were used more frequently in the treatment of KOA and the treatment effects were satisfactory [16, 17]. Therefore, we will carry out a high-quality clinical randomized controlled trial to research the effect laws of these three acupuncture treatments on KOA.

## Methods and analysis

### Study design

**Fig. 1:**
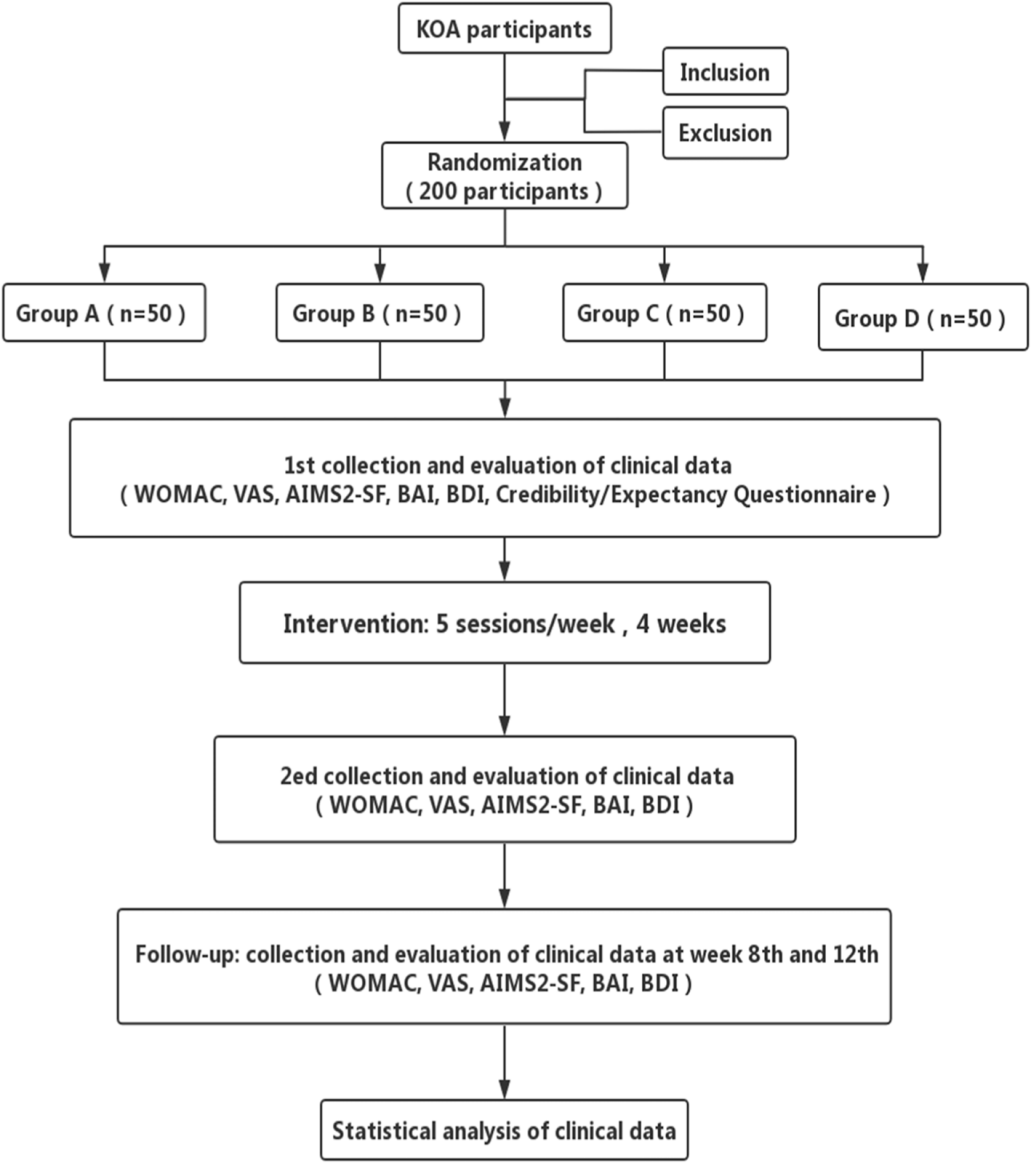
Flowchart of trial procedures. Flowchart of the trial. KOA knee osteoarthritis, WOMAC Western Ontario and McMaster Universities Osteoarthritis Index, AIMS2-SF Arthritis Quality of Life Measurement Scale Simplified Scale, BAI Beck Anxiety Inventory, BDI Beck Depression Inventory, VAS visual analog scale

This is a randomized controlled trial (RCT) that aims to compare the effect of MA, EA, and WA acupuncture methods in the treatment of KOA. This trial has been approved by the ethical committees of the First Affiliated Hospital of Henan University of Traditional Chinese Medicine (No: 2021HL-158) and registered in the Chinese Clinical Trial Registry, ChiCTR2100049526. The protocol will be reported following Standard Protocol Items: Recommendations for Interventional Trials (SPIRIT) reporting guidelines [18] (Additional file 1). A total of 200 patients diagnosed with KOA according to the American College of Rheumatology clinical criteria will be recruited [19]. After informed consent acquisition, all patients will be randomly assigned to group A (MA group), group B (EA group), group C (WA group), and group D (SA group) based on the ratio of 1:1:1:1, to receive treatment. The treatment procedure will include 20 sessions of treatments in total from baseline to week 4 and 2 times of follow-ups on week 8 and week 12 respectively. Outcome measurements will be assessed according to the following time schedule. Figure 1 shows the study design in the flowchart, and Table 1 illustrates the time schedule of enrolment, interventions, assessments, and visits of participants.

**Table 1:** Schedule for enrollment, intervention, and assessments during the study period

Timepoint	Enrollment allocation		Treatment period				Follow-up	
	−1 week	0 week	1 week	2 week	3 week	4 week	8 week	12 week
**Enrolment**								
Eligibility screen	X							
Baseline	X							
Informed consent	X							
Randomization	X							
Medical history	X							
Merger disease	X							
Allocation		X						
**Interventions**								
Group A			X	X	X	X	X	X
Group B			X	X	X	X	X	X
Group C			X	X	X	X	X	X
Group D			X	X	X	X	X	X
**Assessments**								
WOMAC		X				X	X	X
VAS		X				X	X	X
AIMF2-SF		X				X	X	X
BAI		X				X	X	X
BDI		X				X	X	X
CEQ		X						
Adverse events		X	X	X	X	X	X	X

Qualification screening and informed consent will be completed before the assignment. After allocation, each patient will be treated within 4 weeks. The evaluation will be performed at baseline and weeks 4, 8, and 12 respectively after randomization. Adverse events will be recorded on the case report form at any time during treatment

### Study setting and recruitment

The study will be carried out at the First Affiliated Hospital of Henan University of Traditional Chinese Medicine. Two hundred patients with KOA will be recruited via two schemes. The first is to recruit patients in the outpatient and inpatient departments from the First Affiliated Hospital of Henan University of Traditional Chinese Medicine. Moreover, potential patients in local communities and out-hospital clinics will be recruited by advertisements through posters, leaflets, newspapers, and so on. The recruitment information will provide a telephone number so that potential patients can be able to contact the researchers. People willing to participate in the study will be invited to contact the clinical research coordinator (CRC) by telephone. The CRC will perform a preliminary screening of people for inclusion and exclusion criteria and then, if appropriate, schedule a face-to-face baseline visit with a senior specialist in physical and rehabilitation medicine. All eligible patients will provide written informed consent. The CRC will discuss the study in detail (i.e., the study purpose, procedures, and time commitment, as well as the potential risks and benefits associated with participation in the study) with potential patients and obtain written informed consent. The confidentiality of patient records will be protected. At the time of enrolment, each patient will be assigned a unique randomization number, which is the only direct identifier included on all case report forms.

### Participants

#### Inclusion criteria

Participants who meet all of the following requirements will be considered for inclusion: (1) male or female, aged 45–75 years; (2) diagnosed with KOA according to the American College of Rheumatology (ACR) criteria; (3) radiologic confirmation of KOA [20] (Kellgren–Lawrence gradesIto III); (4) the average daily pain scare over 40 points (on a 0- to 100-point scale); (5) symptoms have been present for more than 6 months without redness, swelling, or heat; and (6) signed informed consent.

#### Exclusion criteria

Patients who meet any of the following criteria will be excluded from the trial: (1) are unable to walk; (2) have a serious infection of the knee; (3) have suspected tears in any ligaments or menisci or acute inflammation of the synovial capsule; (4) have a history of trauma, ligament damage, fracture, or surgery on the knee(s) within 6 months, causing pain or functional problems (history of knee replacement will be excluded); (5) have a history of local tumor/malignancy at the knee; (6) have physical or laboratory findings indicating infection, presence of autoimmune disease, or inflammatory arthritis; (7) have knee pain caused by radiculopathy/herniation of an intervertebral disc; (8) have end-stage diseases or other suspected severe conditions such as deep vein thrombosis of the lower limb, edema related to cancer or cancer treatment, severe blood coagulation disorders, uncontrolled systemic arterial hypertension, and severe diabetes; (9) have a history of prolotherapy, hyaluronic acid injections, or corticosteroid injections within 3 months; (10) have received acupuncture, electro-acupuncture, Tui-na therapy, massage, or physiotherapy 8 weeks prior to enrolment in the trial; (11) have severe pain in other regions; (12) have severe mental disorder(s); (13) are oversensitive to needles; and (14) are insensitive to pain due to advanced diabetes, neuropathy, or use of strong painkillers.

### Discontinuation criteria and modification

During the trial period, patients with KOA who meet the following criteria will be excluded from the study: (1) protocol violation such as taking analgesic without permission or receiving additional treatment that may interfere with the efficacy of acupuncture, (2) withdrawal of consent for study participation because the patients do not wish to continue, (3) missing more than 4 of 20 acupuncture treatment sessions, and (4) occurrence of a serious adverse event that the doctors consider should lead to termination of trial participation.

### Randomization

In this trial, eligible patients who consent to participate will be randomly assigned to one of four groups via a central randomization system for clinical research in a ratio of 1:1:1:1 by an independent researcher, who will not be involved in the implementation or statistical analysis of the trial. Random number lists will be created by PROCPLAN of SAS 9.2 (SAS Institute Inc., Cary, NC, USA). The randomization allocation will use sequentially numbered, opaque, and sealed envelopes. The participants’ screening sequence numbers will be printed outside the envelope, whereas the group names will be printed inside. After a participant has met all selection criteria, signed the informed consent form, and completed the baseline assessments, the researcher will inform the statisticians in the center. The statisticians will open the envelope according to the participant’s screening sequence number and then assign the participant to one of the four groups.

### Blinding

Due to the operation characteristics of acupuncture, acupuncturists will be asked to apply the different methods of stimulation, they will not be blinded to treatment allocation for the different locations in each group. However, participants will be blinded to group assignment, and they will be informed that there are four methods of acupuncture treatment provided in this study and will accept one of them in a separate compartment. Each participant will be treated separately to prevent any exchange of study information. In addition, the outcome assessors, data recorders, acupuncturists, and statisticians will all operate independently; the randomization staff and acupuncturist will know the allocation information, while the outcome assessor and statisticians will stay blind to this information throughout the study. Unblinding will not be done until the completion of data analysis, and after the data analysis, we will have a blinded interpretation of the study results to minimize misleading data interpretation. In short, we will implement the three separation principles of researchers, operators, and statisticians for the sake of reducing the risk of bias.

### Sample size

PASS was used for sample size determination. According to the previous study [21] and clinical experience, the success rates of the EA, WA, MA, and SA groups are expected to be 70%, 65%, 60%, and 40%, respectively. A two-sided significance level of 0.05 will avoid the inflation of type I errors. A sample size of 45 patients in each group is estimated to have 80% power to detect significant differences between the groups. To compensate for a 10% loss in follow-up, the sample size was increased to 50 patients in each group.

### Researchers

The acupuncturists who deliver treatments for the four groups are registered with the Ministry of Health of the People’s Republic of China as Chinese medicine practitioners and have more than 6 years of clinical experience. In addition, two supervisory data collectors and analysts who do not know the random assignment protocol will monitor the whole experiment. What is more, people who are responsible for recruiting participants, researchers, outcome assessors, and statisticians in the trial will receive special training regarding the purpose and standard procedure of the project before the trial begins strictly.

### Informed consent

Each patient will be informed of the study protocol information regarding random allocation, possible benefits and risks, and so on. Then, we will provide sufficient time for all patients to decide whether to participate in the trial. Only if they voluntarily sign the informed consent will they be randomized to a group. Moreover, the patients will be free to withdraw from the study at any time without a specific reason and any penalty or loss of benefits. However, we will attempt to record the reason for withdrawal and encourage the participant to remain in the study if possible.

### Safety assessment

Adverse events (AEs) caused by acupuncture such as pain, bleeding, fainting, allergy, or other severe events will be processed immediately and recorded in detail in the case report forms (CRF). Participants with mild and moderate AEs will be treated for their symptoms and closely monitored as necessary by the researcher, while severe AEs will be reported to the Research Ethics Committee, which will provide medical advice to the research team within 48 h, and the Research Ethics Committee will determine whether a termination of the trial is required. Patients may choose to withdraw from the trial due to any AE.

### Interventions

There are 4 groups in this trial, which are group A (MA group), group B (EA group), group C (WA group), and group D (SA group). When experimenting, it is suggested that the patients do not use other methods for the treatment of KOA in addition to the experimental scheme.

#### Group A

**Fig. 2:**
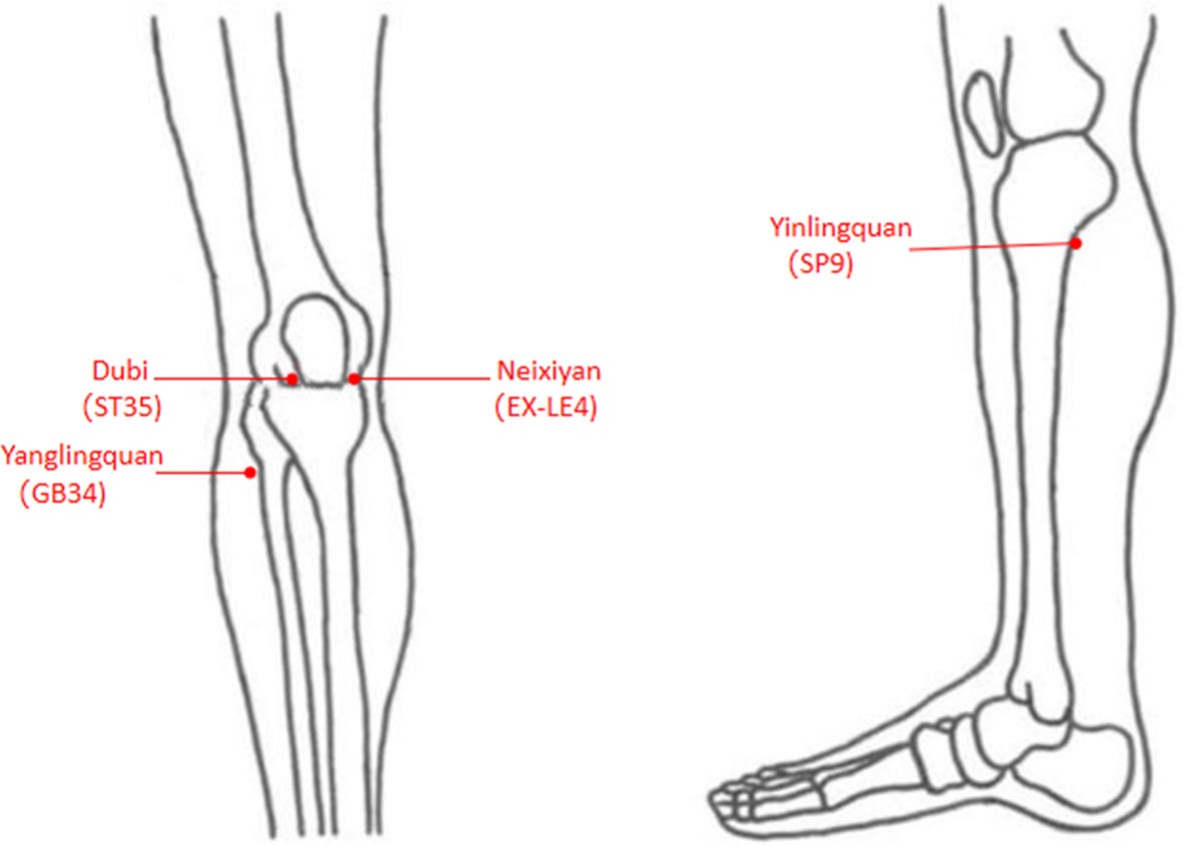
The picture of acupuncture points

Subjects assigned to group A will be treated with MA for 4 weeks. The acupoints used include GB34 (Yanglingquan), SP9 (Yinlingquan), ST35 (Dubi), and EX-LE4 (Neixiyan) (Fig. 2). At the same time, depending on the patient’s condition, two ashi points (the point where the patient feels most pain) can be added. The specific operation method is as follows: after the patients maintain a comfortable position, the acupuncturist will disinfect the skin with 75% alcohol, and use a sterile needle (0.25 mm in diameter, 40 mm in length, Huatuo, Suzhou, China) to the acupuncture points. Needles will be making an optimum insertion into the skin of 21~26 mm. During the acupuncture operation, the acupuncturist will adopt the method of even reinforcing-reducing, while the rotation angle is 90~180°, the lifting amplitude is 0.3~0.5cm and the frequency is 60~90 times/min. After the *deqi* sensation (including soreness, numbness, distention, and heaviness) is obtained, the needle will be retained for 30 min in each session and manipulated every 10 min with intermittent stimulation for maintaining the *deqi* sensation. The needle will be stimulated twice and the manipulation of each acupoint will last for 10 s. MA treatment will be applied once a day, 5 sessions per week for 4 consecutive weeks, and rest for 2 days between treatments.

#### Group B

In group B, subjects will be treated with EA for 4 weeks. The acupoints and acupuncture operation are the same as the MA group, but after *deqi*, an electrical apparatus (HANS-200A acupoint nerve stimulator, Nanjing Jisheng Medical Co, Ltd) will be connected. When both alligator clips are connected to paired needles, it produces electric currents to stimulate acupoints. The electric wave mode will be the dense-sparse wave; sparse wave and dense wave appear alternately, each wave lasting for 1.5 s to avoid body adaptation caused by the continuous output of a single waveform. The power of the electric current will be adjusted to make each acupoint feel the electric stimulation but still comfortable during the treatment process. The needle will be retained for 30 min in each session. EA treatment will be applied once a day, 5 sessions per week for 4 consecutive weeks, and rest for 2 days between treatments.

#### Group C

In group C, subjects will be treated with WA for 4 weeks. The acupoints, location, and acupuncture operation will be the same as those described for the MA group. After *deqi*, acupuncturists will insert a piece of moxa pillar (3 cm) into the end of the needle, and light the tip of the moxa pillar, when the moxa pillar burning is completed, the acupuncturist will remove the ashes. When the time reaches 30 min, the acupuncturist will remove the acupuncture needles and check thoroughly for any possible blisters or bleeding. WA treatment will be applied once a day, 5 sessions per week for 4 consecutive weeks, and rest for 2 days between treatments.

#### Group D

In group D, the non-acupoints used in the SA are all located 1cm away from the outer side of the midline of the lower extremity of the six acupuncture points selected in the MA group. Patients in the SA group will undergo the same procedures as those in the MA group. The needle depth is 1~2mm, and the needle will be directly retained for 30 min without any manipulation in the whole procedure to avoid the *deqi* sensation as much as possible. The SA treatment will be applied once a day, 5 sessions per week for 4 consecutive weeks, and rest for 2 days between treatments.

### Outcome assessments

If only one knee is affected, the assessment of the outcomes will relate to this knee. If the patient has two affected knees and only one meets the ACR criterion, only this knee will be evaluated. In the case that both knees are affected following the inclusion criteria, the more painful knee will be chosen for evaluation.

#### Primary outcome

We will use the Western Ontario and McMaster Universities Osteoarthritis Index (WOMAC) [22] as the primary outcome measure. The WOMAC is a valid and reliable self-administered questionnaire that has been used widely in a variety of clinical trials involving patients with KOA. It can be used as an initial assessment of the intensity of pain and functional disability of KOA. The Chinese version of WOMAC consists of 24 items assessing the patients with KOA pain (5 items), stiffness (2 items), and physical function (17 items). Therefore, it can multidimensionally measure pain, stiffness, and physical functional disability [23]. All 24 questions will be listed on a numerical rating scale ranging from 0 (no symptoms) to 4 (maximum symptoms). Doctors need to help patients perform the self-assessment, collect the total score from the 24 questions, and record it. Higher scores indicate more severe symptoms or physical disability. The evaluation will be performed at baseline and weeks 4, 8, and 12 respectively after randomization; then, we will select the evaluation at week 4 as the primary outcome.

#### Secondary outcome

- ① We will use the Western Ontario and McMaster Universities Osteoarthritis Index (WOMAC) evaluation at weeks 8 and 12 as the secondary outcome measure.
- ② The pain visual analog scale (VAS) [24]: The VAS is an internationally recognized 10-point scale selected to quantitatively evaluate the level of knee pain during the study. It scores from “0 (none),” “about 2 (mild),” “around 5 (moderate),” “almost 8 (severe),” and “nearly 10 (unbearable)” spaced along the continuum. During the experiment, the VAS assessments will be performed at baseline and weeks 4, 8, and 12 respectively by independent assessors who are not involved in acupuncture treatments (asking subjects to indicate the pain intensity at the most painful points during walking and ascending/descending a step and overall).
- ③ Arthritis Quality of Life Measurement Scale Simplified Scale (AIMS2-SF) [25]: We will use the Chinese version of the Arthritis Quality of Life Measurement Scale Simplified Scale by the Fudan University School of Public Health to assess the quality of life. Because this experiment is aimed at KOA patients, 5 items involving hands and arms in the physical dimension of this scale will be removed, and only the remaining 21 items will be used. All items are scored on a scale of 1 to 5, with the total score represented by the sum of 5 dimensions. The higher the score, the better the quality of life. The evaluation will be performed at baseline at weeks 4, 8, and 12 respectively after randomization.
- ④Credibility/Expectancy Questionnaire: The Credibility/Expectancy Questionnaire is a quick and easy-to-administer scale for measuring treatment expectancy and rationale credibility for use in clinical outcome studies [26]. Participants will be asked “How much do you feel acupuncture therapy will help to reduce your symptoms?” at baseline.
- ⑤ Emotional monitoring: Mood changes, such as depression and anxiety, are common concomitant symptoms of KOA. In order to monitor the impact of emotional changes on the condition of KOA, we will use the Beck Anxiety Inventory (BAI) [27] and Beck Depression Inventory (BDI) [28] to evaluate the emotional state of patients in this trial. The evaluation will be performed at baseline and weeks 4, 8, and 12 respectively after randomization.

### Data collecting and monitoring

The governance of this study will be carried out by the trial steering committee (TSC), composed of the primary investigators and statisticians, who will oversee the entire study conduct to ensure that all researchers participating in this study are following the proposed protocol. A monthly meeting will be conducted where the TSC can supervise the progress and the data quality of this study and share suggestions if problems occur. The Ethics Committee will hold an annual meeting to monitor the implementation of the entire trial.

In this study, the data that includes observation time points, scanning time points, outcome measures, adverse events, and safety evaluations will be collected at baseline and weeks 4, 8, and 12 after the intervention using the case report form. And a special assistant will be responsible for reviewing data integrity, accuracy, and consistency. Patients will be assigned to a quiet room for psychological factor assessments. In order to ensure the consistency of source data, all the scales will be assessed by the same researcher during the study. Meanwhile, the research data will be input into EpiData electronic database. For ensuring the accuracy of data entry, data will be entered independently by two researchers, and then the discordances will be resolved by tracing source data.

When the trial is completed, the database will be locked by the data management team, after which the researchers can no longer modify the data. Both paper files and electronic documents will be preserved for at least 5 years after publication. If readers and reviewers have any questions, they can contact the corresponding author for access to the original data. Patient information will remain anonymous, including name, ID number, and telephone number.

### Statistical analysis

Before the data analyses, the research group will provide a statistical scheme to the statisticians. The scheme will include the required data and processing method. The data will be processed and analyzed by the statisticians by the scheme.

Intention-to-treat statistical analysis will be conducted. Missing data will be treated by imputation methods depending on the type: missing completely at random, missing at random, or not at random. Per-protocol analysis will include only patients who attended at least 80% of the sessions and completed the follow-up in the allocated intervention group.

Statistical analyses will be performed with SPSS 22.0 statistics software (IBM Corporation, Armonk, NY, USA). The data analysis process will be completed by statisticians who are independent of the research team and blinded to the test settings. A *t*-test and Wilcoxon test will be used to compare the numerical variables in the within-group analyses, including the WOMAC, VAS, AIMS2-SF, BAI, and BDI. Analysis of variance and the Kruskal-Wallis test will be used for numerical variables in the between-group analyses, including age, height, weight, scores of VAS, WOMAC, AIMS2-SF, BAI, BDI, and Credibility/Expectancy Questionnaire. The chi-squared test and one-way analysis of variance (ANOVA) will be used for categorical variables such as the demographic and baseline characteristics. The available data of the four groups (group A, group B, group C, and group D) will be tested by bilateral tests and the confidence intervals are all 95% bilateral, with a *P* value less than 0.05 being statistically significant.

### Patient and public involvement

Patients and the public were not involved in the development of this clinical trial protocol.

## Discussion

Acupuncture, as a complementary and alternative therapy, is widely used in patients with KOA with the advantages of high safety and fewer side effects, and it becomes increasingly accepted in the world. There is also a growing number of scholars studying on acupuncture for KOA, especially since 2015 when the number of studies has risen sharply [29]. These studies include traditional MA, EA, acupuncture on sensitive acupoints, and thread embedding acupuncture, which fully prove the effectiveness of acupuncture in the treatment of KOA [30–32]. In addition, some scholars have found that the 3 sessions per week of acupuncture work better in improving knee pain and dysfunction than 1 session per week of acupuncture and the benefit of 3 sessions per week persists throughout follow-up [33]. However, the acupuncture treatment method for KOA is still a research topic, particularly the difference in the efficacy of different acupuncture treatments for KOA needs further study. So, this trial is designed as an RCT. The completion of this trial will provide detailed and accurate evidence of the efficacy and safety of MA, EA, and WA on KOA. The advantages of the trial design are shown in the following aspects.

### Choose three classic acupuncture treatments for comparison

The efficacy of acupuncture in the treatment of KOA has been widely recognized internationally. Many organizations, including the Scottish Intercollegiate Guidelines Network [34] and the American College of Rheumatology [14], recommend using acupuncture to treat KOA. Since the invention of the electric acupuncture apparatus, some researchers have also studied the effect of EA on KOA and some studies have shown that EA seems to be better than MA for KOA, but the evidence does not seem to be sufficient [35]. Thus, EA may have a stronger impact on pain and function requires needs to be further confirmed by randomized controlled trials. WA is a characteristic Chinese medicine therapy widely used in clinical practice, which is a kind of therapy combining the advantages of acupuncture and moxibustion. According to the theory of Traditional Chinese Medicine, WA has the efficacy of warming and dredging meridians, regulating blood circulation, and relieving pain [36]. However, to the best of our knowledge, there is no critical appraisal of the evidence for WA in the treatment of KOA. In summary, MA, EA, and WA are all effective at treating pain and dysfunction in patients with KOA. However, few studies directly compare the effects of MA, EA, and WA on KOA. Therefore, this study creatively chooses MA, EA, and WA for comparison.

### Choose appropriate outcome assessments

The Western Ontario and McMaster Universities Osteoarthritis Index (WOMAC) is a valid and reliable self-administered questionnaire that has been used widely in a variety of clinical trials involving patients with KOA. It can be used as an initial assessment of the intensity of pain and functional disability of KOA. The survey shows that WOMAC has high reliability and is relatively simple in evaluating the joint function, pain, and stiffness of KOA patients. It is currently the most popular KOA functional assessment scale in clinical practice. The pain visual analog scale (VAS) is an internationally recognized 10-point scale selected to quantitatively evaluate the level of knee pain during the study. In the clinical evaluation of KOA, VAS is often used in conjunction with WOMAC, and this method is simple and easy and relatively objective and sensitive, as well as the results are intuitive and clear. Arthritis Quality of Life Measurement Scale Simplified Scale (AIMS2-SF) is a scale for measuring the quality of life of KOA patients. The questions in the AIMS2-SF are simple and clear. Patients only need to answer according to the actual situation. The reading level of elementary school or above can be completed by themselves, which is easy to accept for middle-aged and elderly KOA patients. In addition, the test-retest reliability of AIMS2-SF is generally satisfactory and items within the same dimension have high internal consistency. Compared with WOMAC, AIMS2-SF pays more attention to the overall quality of life of KOA patients, so it is widely used to determine the quality of life of KOA patients. KOA is a chronic disease that tends to occur in middle-aged and elderly people. The Credibility/Expectancy Questionnaire is a quick and easy-to-administer scale for measuring treatment expectancy and rationale credibility for use in clinical outcome studies. Due to long-term pain, patients are often accompanied by mood disorders such as depression and anxiety. Therefore, we added the Beck Anxiety Inventory (BAI) and Beck Depression Inventory (BDI) to the outcome assessments to focus on the emotional disturbance of acupuncture treatment for KOA patients. BAI and BDI are simple clinical tools to analyze subjective anxiety and depression symptoms of subjects. They are characterized by concise project content, easy to understand, and easy to operate and analyze.

### Strict quality control

Determining strict control methods is the key to ensuring the methodological quality of RCT. To improve the result reliability of this study, we designed the quality control program from the following aspects: (1) Acupuncture manipulation: The acupuncturists who will participate in the trial have been registered with the Ministry of Health of the People’s Republic of China as Chinese medicine practitioners and have more than 6 years of clinical experience. To avoid the manipulation difference, two veteran acupuncturists, who should receive special and standardized training before the trial, will carry out all the treatment with the standard operating procedure. In addition, superfluous communication between acupuncturists and patients during acupuncture is forbidden. (2) Standardized acupuncture treatment program: The absence of standardized treatment protocol for acupuncture not only obstructs data reproducibility across the discipline but also results in the scientific nature and therapeutic effect of acupuncture not being fully confirmed [16, 17]. In order to make our research reproducible, consistent, and as rigorously scientific as possible, our acupuncture expert panel unanimously approved a standardized acupuncture treatment protocol, which will use fixed and classic acupuncture points (standardized acupuncture with four main points) in patients in the treatment group during every therapeutic session. (3) Multiple separation principle: in this study, all researchers will be trained to receive special training to well understand the design and process of the trial, the use of the CRF, and measurements of quality. What is more, people responsible for recruiting participants, outcome assessors, and statisticians in the trial will not know each other about the grouping of patients. (4) Reduce potential subjective bias: The subjective outcomes may bias the trial results; therefore, we will use the following methods to minimize potential bias: we will train patients so that all patients fully understand the meaning of each scale, and ensure that all patients have the same understanding of the scale without bias. In addition, the outcome assessors will operate independently, they will not be involved in the trial design and statistical analysis, and they are only responsible for explaining the content of the scale to the patients without any inducements. (5) Trial monitoring team: We will set up a trial monitoring team, composed of two supervisory data collectors, investigators, and analysts who do not know the random assignment protocol, and they will oversee the entire study conduct to ensure that all researchers participating in this study are following the proposed protocol. A monthly meeting will be conducted where the trial monitoring team can supervise the progress and the data quality of this study and share suggestions if problems occur.

We will analyze and compare the therapeutic effects of MA, EA, WA, and SA, which will provide not only evidence for different kinds of acupuncture treatments to relieve pain and improve physical function in KOA patients but also visualization reference for the clinical application of acupuncture for KOA management.

### Trial status

The recruitment of patients for this study has not yet begun. The recruitment procedure will begin on September 1, 2021. The authors expect to complete this procedure on January 31, 2023. The protocol (version 1) has been registered at China Clinical Trials Registry on August 2, 2021 (No. ChiCTR2100049526).

### Protocol amendments

If we have amendments to the protocol, the principal investigator will inform the relevant unit and submit a copy of the revised plan to the unit’s office for archiving. At the same time, our research team will also update the protocol of the clinical trial registry.

## Supplementary Information

**Additional file 1.** SPIRIT Checklist for Trials.

## Data Availability

Not applicable. The manuscript does not contain any data.

## Abbreviations

KOA: Knee osteoarthritis
RCT: Randomized controlled trial
MA: Manual acupuncture
EA: Electro-acupuncture
WA: Warm acupuncture
SA: Sham acupuncture
PASS: Power Analysis and Sample Size
SPSS: Statistical Package for the Social Sciences
ACR: American College of Rheumatology
WOMAC: Western Ontario and McMaster Universities Osteoarthritis Index
AIMS2-SF: Arthritis Quality of Life Measurement Scale Simplified Scale
BAI: Beck Anxiety Inventory
BDI: Beck Depression Inventory
CEQ: Credibility/Expectancy Questionnaire
VAS: Visual analog scale
CRF: Case report forms
